# Limited efficacy of cold and heat therapy as adjunctive treatments for local and functional outcomes of *Bothrops atrox* snakebite envenomation: A randomized clinical trial

**DOI:** 10.1371/journal.pntd.0013423

**Published:** 2025-08-14

**Authors:** Mailma Costa de Almeida, Kathleen Maclenny Pereira Carvalho, Yasmim da Silva Mendes, Debora Nery Oliveira, Érica da Silva Carvalho, Marco Aurélio Sartim, Felipe Queiroz Araújo, André Sachett, João Ricardo Nickenig Vissoci, Fernando Almeida-Val, Daniel Barros de Castro, Wuelton Monteiro, Jacqueline de Almeida Gonçalves Sachett

**Affiliations:** 1 School of Health Sciences, Universidade do Estado do Amazonas, Manaus, Amazonas, Brazil; 2 Department of Teaching and Research, Fundação de Medicina Tropical Dr. Heitor Vieira Dourado, Manaus, Amazonas, Brazil; 3 School of Pharmaceutical Science, Universidade Federal do Amazonas, Manaus, Amazonas, Brazil; 4 Duke Department Emergency Medicine and Duke Global Health Institute (DGHI), Duke University, Durham, North Carolina, United States of America; Universidad de Costa Rica, COSTA RICA

## Abstract

**Background:**

*Bothrops atrox* envenomation can cause significant local and systemic effects. Adjunctive therapies, such as cold and heat applications, are proposed to enhance antivenom efficacy, but their clinical value remains unclear.

**Methods:**

This randomized, three-arm clinical trial included 94 patients allocated in a 1:1:1 ratio to Cold Therapy Group (CTG, n = 30), Heat Therapy Group (HTG, n = 31), or Control Group (CG, n = 33). All participants received standard antivenom therapy, with CTG and HTG receiving additional interventions applied for 24 hours post-admission. Primary outcomes included changes in creatine kinase (CK) levels. Secondary outcomes assessed pain intensity, edema, local temperature, and functional recovery using the World Health Organization Disability Assessment Schedule (WHODAS 2.0) assessed four to six months after hospital discharge. Kaplan-Meier survival analysis evaluated time-to-event outcomes.

**Findings:**

Baseline characteristics were comparable across groups. CK levels decreased similarly in all groups at 48 hours (*p* = *0.89)*. No significant differences were observed in the reduction of limb circumference, edema extent and bite site temperature, either the ITT or PP analysis. CTG showed a significant reduction in pain within 24 hours in the per-protocol analysis (Log-rank *p* = *0.04*). Disability assessed by WHODAS 2.0 revealed no significant differences between groups after 6 months of follow-up. No adverse events were associated with the interventions.

**Interpretation:**

Adjunctive HTG had no efficacy in treating local effects of *B. atrox* envenomation. Adjunctive CTG demonstrated benefits observed in pain reduction.

## Author summary

Snakebite envenomation caused by *Bothrops atrox* is a significant health issue in the Amazon region, where access to healthcare is often limited. Antivenom therapy is effective in neutralizing systemic venom effects and may also help reduce local damage, such as pain, swelling, and muscle injury. However, local symptoms often persist, and accessible, low-cost adjunctive therapies could play a complementary role in improving patient care. In this study, we evaluated whether cold and hot compresses, when used alongside antivenom therapy, could further reduce local effects of envenomation. We conducted a clinical trial in the Brazilian Amazon involving 94 patients who received antivenom therapy and were randomly assigned to one of three groups: cold therapy, hot therapy, or control (antivenom therapy alone). While cold compresses helped reduce pain during the first 24 hours, neither cold nor hot compresses had a significant impact on swelling and muscle damage. These findings suggest that cold therapy offer early pain relief. This study highlights the need for further research on supportive care for snakebite patients.

## Introduction

Snakebite envenomation is an outdated disease associated with poverty and predominantly affecting rural and economically disadvantaged environments (1,2). According to the World Health Organization (WHO), these events cause significant socioeconomic impacts in high incidence areas (3–5). In the Brazilian Amazon region, *Bothrops atrox* is the most common venomous snake, responsible for 90% of recorded cases, and is found mainly in forests, but is also found in agricultural and urban areas [1,2].

In Brazil, especially in the Amazon region, *B. atrox* venom exhibits proteolytic, coagulant, hemorrhagic, myotoxic, edematogenic, and hemolytic activities [3]. These venom activities lead to extensive local and systemic effects, with local tissue damage being a major contributor to morbidity. Snake venom metalloproteinases (SVMPs) and phospholipases A2 (PLA2s) are the principal components responsible for the local effects, including edema, necrosis, and hemorrhage [4]. The action of *B. atrox* venom initiates a rapid and intense local inflammatory response characterized by hemorrhage, pain, myonecrosis, edema, and a marked leukocyte inflammatory infiltrate in the affected tissues [5,6].

Antivenom therapy is the recommended treatment for these envenomations, demonstrating high efficacy against circulating venom and partial neutralization of local lesions and complications associated with envenomation [7,8]. There is a need to explore complementary therapies that improve antivenom treatment in reducing local tissue damage [9]. Studies have shown that heat and cold therapies offer distinct benefits in different contexts. Animal models have shown that heat can improve mobility and alleviate discomfort after envenomation, although it can intensify muscle necrosis in some cases, while cold is safe and does not aggravate injuries [10]. Heat and cold can affect inflammation and pain following envenomation. While animal models have shown modulation of tissue damage with these therapies [9], in humans, cold therapy has limited clinical benefit as adjuvant to antivenom treatment [11]. This is the first randomized clinical trial to compare the use of heat and cold therapies as adjuvant therapies in the treatment of *Bothrops atrox* envenomation.

In humans, cold therapy has been shown to be more effective in reducing pain and swelling and in aiding functional recovery, especially in cases of musculoskeletal injuries and after surgery. On the other hand, heat has better results over prolonged periods, promoting greater blood circulation and relieving persistent muscle pain [12]. Trials in specific conditions, such as osteoarthritis and primary dysmenorrhea, also reinforce that cold has more immediate effects in controlling pain and inflammation, while heat is more indicated for muscle relaxation and prolonged recovery [13]. Thus, both approaches are effective and should be applied according to the type of injury and the stage of treatment [10].

This study aimed to evaluate the effectiveness of cold and hot therapies as adjunctive treatments alongside antivenom therapy for managing local tissue damage caused by *B. atrox* envenomation.

## Methods

### Ethics statement

The study was conducted in compliance with ethical standards and the principles outlined in the Declaration of Helsinki. Ethical approval was obtained from the Ethics Committee of the FMT-HVD, with protocol approval number 55959922.1.0000.0005. The trial was registered with the Brazilian Clinical Trials Registry (ReBec) under the number UTN: U111-11691005. All participants provided written informed consent before enrollment. The consent process included a thorough explanation of the study’s purpose, procedures, potential risks, and benefits. All study interventions, including antivenom therapy and adjunctive treatments, were provided at no cost to participants. Confidentiality was maintained throughout the study, with all patient data anonymized and securely stored in a password-protected database accessible only to authorized research personnel.

### Study design and setting

This was an open-label, randomized, three-arm parallel clinical trial conducted from June 2021 to December 2023. Participants were allocated in a 1:1:1 ratio to one of three groups: Cold Therapy combined with antivenom treatment, Heat Therapy combined with antivenom treatment, or antivenom treatment alone (control). The study aimed to evaluate the efficacy of Cold Therapy and Heat Therapy as adjunctive treatments in reducing muscle damage and local inflammatory symptoms (pain, edema, and temperature) in patients with *B. atrox* envenomation. Through a randomized clinical trial conducted in the Brazilian Amazon, the study assessed outcomes such as reductions in creatine kinase (CK) levels, pain intensity, and edema. These findings provide evidence on the potential role of thermal therapies in improving supportive care for snakebite envenomation in resource-limited settings.

The trial was conducted at the Fundação de Medicina Tropical Dr. Heitor Vieira Dourado (FMT-HVD) in Manaus, Brazilian Amazon. FMT-HVD is a tertiary care hospital specialized in the treatment of tropical diseases, including those caused by venomous animals. Located in a region with one of the world’s highest incidences of snakebites, the hospital provided access to a representative population of *Bothrops* envenomation cases. Its facilities supported comprehensive care, including antivenom administration, clinical monitoring, and the expertise required to manage venom-induced complications. This setting ensured the feasibility of the trial, and the reliability of clinical assessments performed during patient hospitalization.

### Sample calculation

The sample size calculation determined that a minimum of 63 patients (21 per group) was required for the Per Protocol (PP) analysis. To account for potential protocol deviations and dropouts, 94 patients were included in the Intention to Treat (ITT) analysis. This sample size provided a statistical power of 90% (*β = 0.10*) to detect main effects (group, assessments, and time) and their interactions, with a significance level of 5% (*α = 0.05*). The estimated effect size (*w = 0.50*) was based on a 50% improvement in baseline myonecrosis (D0), derived from previous local studies using laser therapy [14] for *Bothrops* envenomation. The calculation was performed using G*Power software, ensuring an adequately powered study to detect meaningful clinical differences.

### Participants, recruitment, randomization

Patients with confirmed *Bothrops* envenomation were recruited for the study upon presentation to the FMT-HVD during the study period. Diagnosis was based on clinical and epidemiological features, such as the presence of local swelling, pain, and systemic symptoms typical of *Bothrops* envenomation. When possible, identification of the responsible snake was conducted by a biologist specializing in venomous animals, particularly if the snake was brought by the patient.

Inclusion criteria included patients aged 18 years or older, presenting with signs of *Bothrops* envenomation within 24 hours of the snakebite, and willing to provide informed consent for participation in the trial. Exclusion criteria included delayed presentation beyond 24 hours post-bite, snakebites caused by other species, pre-existing medical conditions that could interfere with study outcomes (e.g., chronic inflammatory or autoimmune disorders), or refusal to participate.

Eligible patients were screened upon arrival at the hospital. After verifying their eligibility, informed consent was obtained prior to randomization. Recruitment was conducted in a consecutive manner to ensure all eligible patients were offered the opportunity to participate.

**Fig 1:**
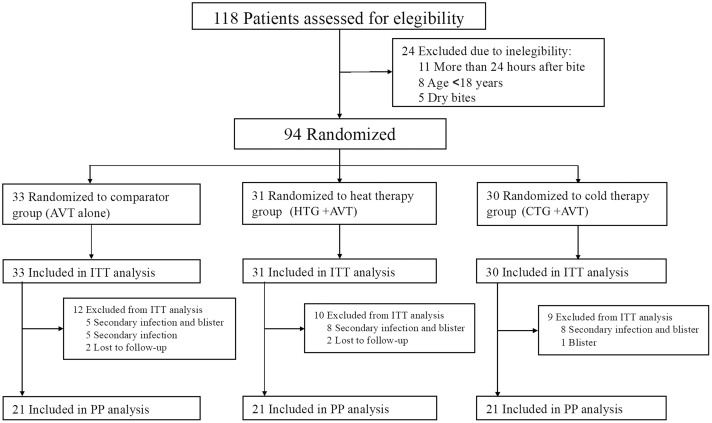
Flowchart for the inclusion of study participants here. AVT indicates antivenom treatment; CTG – Cold Temperature Group; HTG – Hot Temperature Group; CG – Control Group.

Participants were randomly assigned to one of three intervention groups: Cold Therapy Group (CTG), Heat Therapy Group (HTG), or Control Group (CG). Randomization was performed using a computer-generated sequence to ensure unbiased allocation, with a 1:1:1 ratio between the three groups. The randomization process was managed independently by a member of the research team who was not involved in patient care or data collection, minimizing potential allocation bias (Fig 1).

### Intervention

Snakebite treatment in Brazil is guided by a strict protocol established by the Ministry of Health, which specifies the use of antivenom therapy (AVT) adapted to the snake species involved, such as *Bothrops* or *Bothrops-Lachesis*. To ensure appropriate treatment, snakebite cases are classified into three categories based on clinical severity. Mild cases are characterized by localized symptoms, including pain, edema, and bruising at the bite site. Moderate cases present with more severe local manifestations, such as coagulopathy and mild bleeding, but without necrosis or shock. Severe cases are fatal, involving complications such as severe bleeding, renal failure, and systemic symptoms.

All patients in this study received a single premedication dose of 500 mg intravenous hydrocortisone and 5 mg oral dexchlorpheniramine, administered according to institutional standards to reduce the risk of adverse reactions prior to AVT infusion. Antivenom was administered intravenously over 35–45 minutes in 100 milliliters of 0.9% saline solution. Dosages were adapted to the severity of envenomation following the protocols of the Ministry of Health. In addition to antivenom, pain management was implemented for all patients. Metamizole (1 gram orally every 6 hours) was used as a first-line analgesic. For patients with severe or refractory pain not adequately relieved by metamizole, 100 mg of intravenous tramadol was administered as needed.

Initial cleaning of the snakebite site was performed using 0.9% saline solution. Soap and water were used when necessary to remove dirt or debris. After cleaning, the bite site was assessed in detail.

In the CTG and HTG groups, thermal therapies were applied to the bite site using sterile gauze and thermal bags maintained within the target temperature range of 10–15°C (CTG) or 40–45°C (HTG). Thermal bags were applied for 20 minutes every 12 hours during the first 72 hours of hospitalization. The application area was defined using infrared thermography, which indicated the extent of local inflammation for application to the entire affected area. Patients in CG received standard antivenom therapy without any additional thermal intervention.

All interventions were conducted under the supervision of trained healthcare professionals. Due to the nature of the interventions, the study was conducted as an open-label trial. Participants and investigators were aware of group assignments, as blinding was not feasible. However, all clinical assessments, including measurement and documentation of outcomes, were performed by trained healthcare professionals following standardized protocols to reduce potential observer bias.

### Baseline data

Data collection was conducted at the bedside during participants’ hospitalization to ensure accurate and timely documentation of clinical, laboratory, and procedural information. Trained healthcare professionals used standardized forms to document baseline characteristics, outcome measures, and adverse events. Predefined time points for data collection included baseline (D0), 24 hours (D1), and 48 hours (D2).

Baseline information recorded included the time elapsed from the bite to medical care (in hours), the time spent walking after the bite (in minutes), history of previous snakebites, and the use of pre-hospital medications, either described or taken orally. Additional data included the use of a tourniquet, and any other pre-hospital therapies administered. Laboratory measurements included creatine kinase (CK) levels, blood cell counts, platelet counts, clotting time (CT) and prothrombin activity time (PT) and C-reactive protein (CRP).

Outcome measures were documented following standardized protocols. Pain intensity was recorded using a Visual Analog Scale (VAS), while edema was assessed by measuring the circumference of the affected limb at a standardized distance from the bite site. The extent of edema in the affected limb was assessed by measuring the distance between the distal and proximal points exhibiting swelling. Proportion of circumference measurement (bite site circumference/ circumference of the contralateral limb), 48 hours after admission. Local temperature readings were obtained using an infrared thermometer, and functional disability was assessed using the World Health Organization Disability Assessment Schedule (WHODAS 2.0). WHODAS 2.0 assessments were conducted via telephone interview 4–6 months after patient discharge. Data were securely stored in a password-protected database accessible only to authorized members of the research team.

### Feasibility outcomes

We obtained patient eligibility rates, recruitment rates and retention rates. Retention rate was calculated in two ways per protocol (48 hours after admission, which is the time point at which the primary outcome was measured) and by intention to treat, and WHODAS 2.0 4–6 months after discharge of patients, to determine the long-term disability outcome in this study. Data on fidelity to the treatment protocol were obtained by measuring the proportion of interventions that needed adaptations/changes out of the total number of interventions. Our aim was to assess whether fidelity was a potential issue in this trial. For feasibility, we measured the feasibility of the trial design by deviations from the trial protocol in relation to data collections.

### Safety outcomes

During and after the interventions, local adverse effects were monitored, including cold and heat burns, dryness or irritation of the skin [15,16].

### Efficacy outcomes

Primary Outcomes: The primary outcome was the reduction in creatine kinase (CK) levels, a biomarker of muscle damage, measured at baseline (D0), 24 hours (D1), and 48 hours (D2) after the start of treatment.

Secondary Outcomes: Secondary outcomes included pain intensity, edema, local temperature, and disability assessment. Pain intensity was evaluated using a Visual Analog Scale (VAS), where patients rated their pain on a scale from 0 (no pain) to 10 (worst pain). Edema was assessed by measuring the circumference of the affected limb at a standard point relative to the bite site, using a measuring tape for consistent measurements. Local temperature at the envenomation site was recorded using a contact thermometer, ensuring uniform application for accurate readings. All outcomes were recorded by trained healthcare professionals at the predefined time points (D0, D1, and D2).

Disability was assessed using the World Health Organization Disability Assessment Schedule (WHODAS 2.0), which evaluates six domains of functioning: cognition, mobility, self-care, getting along, life activities, and participation. WHODAS scores were collected at baseline and at 48 hours to capture the short-term functional impact of envenomation and the interventions.

### Statistical analysis

The analysis was conducted using both the Intention to Treat (ITT) and Per Protocol (PP) datasets. Descriptive statistics were used to summarize baseline characteristics, with continuous variables expressed as medians and interquartile ranges, and categorical variables presented as absolute numbers and percentages. The Shapiro-Wilk test was used to assess data normality. Median values for creatine kinase activity, pain intensity, proportional differences in limb circumference, extent of edema, and the temperature difference between the bite site and the contralateral limb were compared using the Kruskal-Wallis rank-sum test. Bonferroni correction was applied to adjust for multiple pairwise comparisons. Kaplan-Meier survival analysis was performed to evaluate the time from admission to achieving a 50% reduction in CK levels. Differences between groups (CTG, HTG, and CG) were assessed using the Log-rank test, and survival curves were generated to visualize the results. All statistical analyses were conducted using R software, within the RStudio IDE, version 4.1.2 (Posit PBC). Statistical significance was set at *p < 0.05* for all tests, and missing data were addressed using multiple imputation methods to ensure the robustness of the results.

## Results

### Characterization of the participants.

A total of 94 patients were randomized into three groups: Cold Therapy Group (CTG, n = 30), Heat Therapy Group (HTG, n = 31), and Control Group (CG, n = 33). Baseline characteristics, including epidemiological, clinical, and laboratory parameters, were comparable across groups, with no significant differences observed (Table 1). The median time from the snakebite to hospital admission was 3.7 hours (IQR: 1.6–8.5, *p = 0.96*). Most participants were male (88.3%) and resided in rural areas (69.1%). Moderate envenomation was the most common presentation (58.5%, *p = 0.69*).

**Table 1:** Epidemiological, clinical, and laboratory characteristics of study participants.

Characteristic	Total	CG	CTG	HTG	Adjusted p-value
	(N = 94)	(N = 33)	(N = 30)	(n = 31)	
**Sex**					
Male	83 (88.3)	31 (93.9)	25 (83.3)	27 (87.1)	0.69^2^
**Residence**					
Rural	65 (69.1)	22 (66.7)	22 (73.3)	21 (67.7)	0.95^1^
Urban	29 (30.9)	11 (33.3)	8 (26.7)	10 (32.3)	
**Age group (years)**					
18–30	22 (23.4)	7 (21.2)	5 (16.7)	10 (32.2)	0.69^2^
31–40	22 (23.4)	8 (24.2)	6 (20.0)	8 (25.8)	
41–50	18 (19.1)	3 (9.1)	8 (26.7)	7 (22.6)	
51–60	15 (16.0)	5 (15.2)	7 (23.3)	3 (9.7)	
>60	17 (18.1)	10 (30.3)	4 (13.3)	3 (9.7)	
**Bite site**					
Hand	16 (17.0)	7 (21.2)	6 (20.0)	3 (9.7)	0.85^2^
Foot	60 (63.8)	20 (60.6)	20 (66.7)	20 (64.5)	
Leg	16 (17.0)	6 (18.2)	3 (10.0)	7 (22.6)	
Ankle	2 (2.2)	0 (0.0)	1 (3.3)	1 (3.2)	
**Time to treatment (h, IQR)**	3.7 (1.6-8.5)	3.3 (1.7-9.7)	2.9 (1.2-6.4)	4.3 (1.8-8.7)	0.96^3^
**Previous snakebite**	13 (13.8)	7 (21.2)	3 (10.0)	3 (9.6)	0.69^2^
**Use of topical medicines**	35 (37.2)	13 (39.4)	6 (20.0)	16 (51.6)	0.66^1^
**Severity classification**					
Severe	19 (20.2)	6 (18.2)	7 (23.3)	6 (19.3)	0.69^2^
Mild	20 (21.3)	9 (27.3)	8 (26.7)	3 (9.7)	
Moderate	55 (58.5)	18 (54.5)	15 (50.0)	22 (71.0)	
**Analgesic medications used**	58 (61.7)	21 (63.6)	18 (60.0)	19 (61.3)	0.66^1^
Dipyrone	36 (62.1)	14 (66.6)	13 (72.2)	9 (47.4)	0.66^2^
Dipyrone plus tramadol	10 (17.2)	6 (28.6)	1 (5.6)	3 (15.7)	
Paracetamol	1 (1.7)	0 (0.0)	0 (0.0)	1 (5.3)	
Tramadol	11 (19.0)	1 (4.8)	4 (22.2)	6 (31.6)	
**Systemic manifestations**					
Headache	29 (30.9)	8 (24.2)	13 (43.3)	8 (25.8)	0.69^1^
Gum bleeding	9 (9.6)	2 (6.1)	5 (16.7)	2 (6.5)	0.69^2^
Vomiting	10 (10.6)	2 (6.1)	6 (20.0)	2 (6.5)	0.66^2^
Nausea	19 (20.2)	7 (21.2)	9 (30.0)	3 (9.7)	0.66^1^
**Secondary infection**	19 (20.2)	9 (27.3)	5 (16.7)	5 (16.1)	0.45^1^
**Local bleeding**	12 (12.7)	5 (15.1)	5 (16.6)	2 (6.4)	0.70^2^
**Clotting time**					
Non-coagulable	60 (63.8)	18 (54.5)	19 (63.3)	23 (74.2)	0.69^1^
**Prothrombin time**					
Abnormal (>14s)	79 (84.9)	26 (81.3)	27 (90.0)	26 (83.9)	0.87^2^
**C-reactive protein (> 6.5 mg/dL)**	26 (31.3)	9 (32.1)	9 (33.3)	8 (28.6)	0.95^1^
**Leukocytes (IQR, x10** ^**3**^ **/mm3)**	12.1 (8.3-15.3)	11.6 (8.1-15.3)	10.9 (8.4-15.3)	12.3 (8.3-14.3)	0.96^3^
**Platelets (IQR, x10** ^**3**^ **/mm3)**	214 (181-247)	210 (183-234)	209 (174-261)	227 (185-249)	0.96^3^
**Hemoglobin (IQR, g/dL)**	15.3 (14.3-16.5)	15.7 (14.1-16.7)	14.9 (13.8-16.0)	15.2 (14.7-16.6)	0.96^3^

CTG – Cold Therapy Group; HTG – Heat Therapy Group; CG – Control Group.

Reference values: Leukocytes: 4–10 x10^3^/mm3 Platelets: 150–450 x10^3^/mm3; Hemoglobin: 12.5–15.5 g/dL for and 12.0–14.0 for females; Clotting time: 4–10 minutes; Prothrombin time: 10–13 seconds; C-reactive protein: < 6.5 mg/dL. IQR - interquartile range.

^1^Chi-squared test

^2^Fisher exact test

^3^Kruskal-Wallis

### Feasibility, fidelity, and safety outcomes

Out of 118 patients assessed for eligibility, 94 (79.6%) met the inclusion criteria and were randomized. The ITT analysis included all 94 patients, while 63 patients (67%) completed the protocol and were included in the PP analysis. Fidelity to the intervention protocol was maintained across all groups, with no modifications or deviations.

Regarding feasibility, no protocol deviations were observed due to protocol breakage in terms of data collection. No patient reported any discomfort during the application of cold and hot therapies or expressed that they would like to withdraw from the study because of any procedure performed. Regarding safety outcomes, no adverse events were observed in any of the study groups.

### Efficacy outcomes

#### Primary outcome.

At admission, median CK levels were comparable across groups (*p = 0.33*). After 48 hours, CK levels decreased in all groups, with no significant differences between groups (median reductions: CTG 4.68 U/L, HTG 4.73 U/L, CG 4.77 U/L; *p = 0.89*). The reductions in CK levels across groups are summarized in Table 2.

**Table 2:** Efficacy primary and secondary outcomes.

Outcomes	At admission				48 h			
	CG (n = 33)	CTG (n = 30)	HTG (n = 31)	*p- value* ^*a*^	CG (n = 33)	CTG (n = 30)	HTG (n = 31)	*p-value*
Primary outcome								
Creatine kinase, (median, IQR, U/L)	229.5 (239.2)	194.5 (125.7)	171.0 (184.5)	*0.33*	116.5 (311.0)	106.5 (126.2)	113.0 (105.7)	*0.89*
Secondary outcomes								
Pain intensity^b^	6 (4)	7 (4)	6 (3)	*0.89*	3 (5)	2 (4)	3 (4)	*0.26*
Limb circumference measurement ratio^c^, %	11.1 (8.6)	11.1 (4.9)	8.70 (6.1)	*0.15*	6.49 (8.9)	9.09 (9.0)	8.11 (4.3)	*0.43*
Extent of edema in affected limb^d^, cm	47 (32.0)	29 (29.0)	38 (24.0)	*0.22*	46.5 (43.8)	30 (34.0)	42 (37.0)	*0.18*
Difference between bite site temperature and that of contralateral limb^e^, °C	0.6 (1.3)	1.4 (2.8)	0.4 (1.7)	*0.02*	1.25 (1.6)	1.5 (2.3)	0.9 (2.6)	*0.24*

CTG – Cold Therapy Group; HTG – Heat Therapy Group; CG – Control Group.

Reference values: Creatine kinase: 24–190U/L.

^a^Comparison of groups was done by the Kruskal-Wallis rank-sum test, with Bonferroni correction.

^b^Pain assessment was carried out using the numerical rating scale, with values ranging from 1 to 10.

^c^Circumference measurement ratio (circumference of the bitten limb/circumference of the contralateral limb).

^d^Extent of edema in the affected limb evaluated by measuring the distance between the distal and proximal points showing swelling.

^e^Difference between temperature of the bite site and that of the corresponding site on the contralateral limb

**Fig 2:**
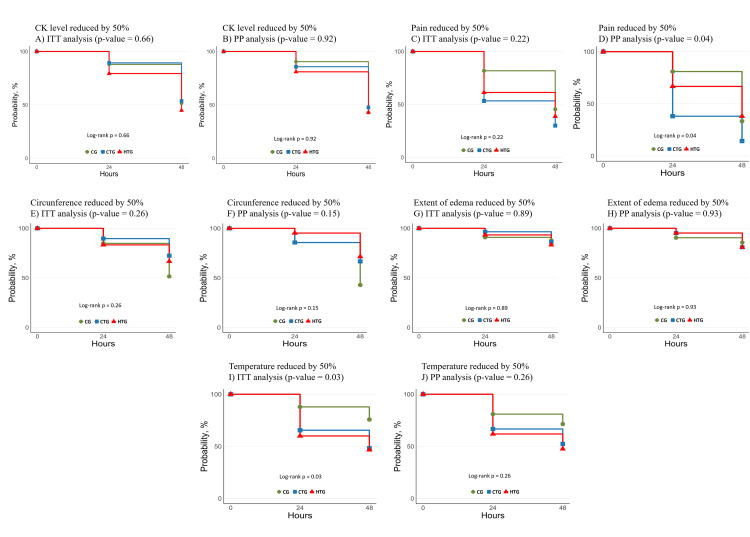
Kaplan-Meier survival curves for primary and secondary outcomes in ITT and PP analyses. **(A)** Time to 50% reduction in creatine kinase (CK) levels in the Intention to Treat (ITT) analysis. **(B)** Time to 50% reduction in CK levels in the Per Protocol (PP) analysis. **(C)** Time to 50% reduction in pain intensity in the ITT analysis. **(D)** Time to 50% reduction in pain intensity in the PP analysis. **(E)** Time to normalization of temperature difference (bite site versus contralateral limb) in the ITT analysis. **(F)** Time to normalization of temperature difference in the PP analysis. Survival curves compare outcomes across the Cold Therapy Group (CTG), Heat Therapy Group (HTG), and Control Group (CG). Log-rank tests were used to assess statistical significance for differences between groups in all panels. Hazard ratios and statistical results are presented in the respective text and tables. Each panel illustrates the cumulative proportion of participants achieving the specified outcome over time, with confidence intervals shaded where applicable.

Kaplan-Meier survival analysis revealed no significant differences in the time to achieving a 50% reduction in CK levels across groups in either the ITT analysis (Log-rank *p = 0.66*, Fig 2A) or PP analysis (Log-rank *p = 0.92*, Fig 2B).

#### Secondary outcomes.

The median pain scores at the time of admission for the CG, CTG, and HTG groups were 6, 7, and 6, respectively (*p = 0.89*). A reduction in pain scores was observed in all analyzed groups after 48 hours (*p = 0.18*). However, in the survival analysis, the group that received cold therapy achieved a 50% reduction in pain in the first 24 hours, with *p = 0.04*, which was not the case in the other groups. In this analysis, cold therapy was more effective than hot therapy for pain control. Regarding the assessment of limb circumference at the time of admission, there were no significant differences among the groups (*p = 0.15*), nor was there a significant difference after 48 hours. Similarly, the extent of edema did not show a significant difference between the groups after 48 hours (*p = 0.18*). At the time of admission, the temperature at the bite site was higher than that of the contralateral area. After 48 hours, the temperature difference was even more pronounced, with no significant differences observed among the groups (*p = 0.24*), as presented in Table 2.

Changes over time as shown in Fig 2, changes over time illustrate the difference related to the duration from admission to the day a 50% reduction in outcomes was achieved among the intervention and control groups. No differences in the time to reduction of creatine kinase (CK) levels were observed between the analyzed groups in both the ITT population and the PP population.

Regarding pain, while no differences were evident in the ITT analysis, a reduction in time was noted for the group that applied ice to the site compared to the CTG and CG groups in the PP analysis (*p = 0.04*). As for limb circumference and edema extent, no differences in the time to reduction were observed in either the ITT or PP groups. The time to reduction in temperature was shorter in the ITT group (Log-rank *p = 0.03*) compared to the PP group (Log-rank *p = 0.30*).

### Safety outcomes

No adverse effects were identified as being caused by the cold and heat therapy.

### Functionality outcomes

The analysis of the WHODAS 2.0 domains revealed no significant differences between the thermotherapy groups and the control group, both in the individual domains and in the summary scores. This indicates that thermotherapy did not have a noticeable impact on the functionality or disability of participants compared to the control group, as shown in Table 3.

**Table 3:** WHODAS Outcomes (ITT Analysis).

Disability assessment	CG (n = 22)	CTG (n = 21)	HTG (n = 18)	*p-value* ^*a*^
Cognition (domain 1)	0 (0)	0 (5)	0 (10)	*0.89*
Mobility (domain 2)	0 (12.5)	0 (18.8)	6.25 (17.2)	*0.37*
Self-care (domain 3)	0 (0)	0 (0)	0 (0)	*0.95*
Getting along (domain 4)	0 (0)	0 (8.3)	0 (8.3)	*0.60*
Life activities (domain 5)	0 (0)	0 (8.3)	0 (14.6)	*0.45*
Social participation (domain 6)	0 (3.1)	0 (8.3)	4.17 (12.5)	*0.18*
Summary score	0.5 (6.1)	1.9 (9.4)	3.8 (13.2)	*0.42*

CTG – Cold Temperature Group; HTG – hot temperature group; CG – control group.

^a^Comparison of groups was done by the Kruskal-Wallis rank-sum test, with Bonferroni correction.

## Discussion

Ancillary therapies of local manifestations of *Bothrops* envenomations are challenging due to the immediate toxicity of the *Bothrops* venom, which causes direct and indirect injury by the presence of venom toxins, and subsequent inflammatory reaction [17–21]. Preclinical studies have shown the efficacy of anti-inflammatory drugs [22] and toxin inhibitors [23] in snake envenomation. A clinical trial showed promising results of low-intensity laser photobiomodulation in *B. atrox* envenomations [14]. In snakebites, the use of thermal therapy is still empirical and clinical trials are necessary to estimate its efficacy. This study aimed to evaluate the efficacy of adjunctive thermal therapies (cold and heat compress) combined with standard antivenom treatment for *Bothrops* envenomation.

Our findings demonstrate that thermal therapies did not significantly outperform antivenom therapy alone in most outcomes. Regarding CK levels evolution, the primary outcome of this trial, there was no difference between study arms, which shows a lack of benefit from thermal therapies on myonecrosis. Both heat and cold therapy did not significantly reduced muscle necrosis of rats experimentally inoculated with *Agkistrodon piscivorus* venom [9]. Moreover, adjunctive cold therapy was not effective in edema recovery in a cases series in Mexico [11]. Several studies indicate a lack of effectiveness of cold therapy in recovering from muscle injuries [24–26], and in sports medicine there is evidence that cold therapy can harm muscle regeneration in athletes [27,28]. In animal models, the results are controversial, depending on the cooling method used, the time and repetitions of applications, and concomitant therapies. A single 20-minutes application of ice immediately after muscle injury is detrimental to muscle regeneration after crush injury, delaying recovery and promoting fibrosis [29]. Prolonged cryotherapy (6 hours) demonstrated beneficial effects in reducing microcirculatory dysfunction, regional inflammation and muscle necrosis in an experimental model of traumatic soft tissue injury in rats [30]. Heat therapy had no significant impact on muscle regeneration [31,32].

Edema is a common clinical manifestation in cases of *Bothrops* envenomation [33]. Its presentation may vary in volume and extent, ranging from localized swelling at the bite site to involvement of the entire limb [33]. In Brazil, the extent of edema is a key criterion used to assess the severity of envenomation and determine the appropriate dosage of antivenom to be administered [34]. In the present clinical trial, no significant differences in edema were observed between heat and cold therapies in this trial, suggesting that while both methods have potential applications in various clinical contexts, their impact on snakebite-induced edema is limited. In other acute injuries, cold therapy generally had a beneficial effect in reducing inflammation [24,26,27]. Otherwise, use of heat therapy requires caution in acute inflammatory diseases, such as arthritis and bursitis, because increased blood flow can lead to fluid accumulation, worsening inflammation and increasing the risk of complications [35–37].

It has long been known that the temperatures used in thermal therapy have no influence on the coagulant and proteolytic actions of *Bothrops* venom [38]. From a point of view of the pathophysiology of *Bothrops* snakebites, it could be hypothesized that the snakebite patients could benefit from both cold and heat therapies. Cold-induced vasoconstriction limits blood flow and the extravasation of inflammatory mediators, and decreases cellular metabolism, which contributes to the control of the inflammatory response and tissue injury [39–41]. On the other hand, heat promotes vasodilation, improves blood circulation, tissue oxygenation and removal of catabolites [15,42–44], and possibly antivenom bioavailability in the bite site. The blood kinetics of snake venom typically follows a two-compartment model, which includes a rapid distribution phase followed by a slower elimination phase, with bioavailability of venom toxins reaching up to 81.5% [45]. *Bothrops* venom remains in the extravascular space, such as in blister exudates, for days after the bite, acting directly on the extracellular matrix and stimulating an intense release of pro-inflammatory mediators [46–49]. In the case of cold therapy, vasoconstriction is not an adequate way to prevent the progression of the inflammatory response in an environment in which toxins are concentrated, sustaining the tissue damage. The prolonged presence of venom in the extravascular space and release of damage-associated molecular patterns (DAMPs) also explains the limited efficacy of antivenom in controlling local manifestations of *Bothrops* envenomation [47,48,50,51]. In turn, the increase in blood flow to the bite site during the heat therapy, at least at the temperature used in this study, apparently is not capable of clearing toxins concentrated in the extravascular space.

Cold therapy demonstrated an advantage in achieving a 50% reduction in pain intensity over time in the PP analysis. This results was not evident in the ITT analysis, possibly because secondary bacterial infection increases the painful sensation in *Bothrops* snakebites, influencing the results [5]. The beneficial analgesic effect in the absence of secondary infection was previously observed on musculoskeletal injuries or neuropathic pain, where painful stimuli are intense and sustained [13,40,41,52–54]. The observation of a modest analgesic effect in this study, despite the absence of a significant edema reduction effect, indicates that this benefit is due to the action of cold therapy reducing nerve impulse conduction through the gate control system [39]. Although overall pain reduction at 48 hours was similar among groups, survival analysis revealed that patients in the CTG group experienced a 50% reduction in pain within the first 24 hours. This early improvement occurred despite similar analgesic use across groups, suggesting that cold therapy may have contributed to a faster initial analgesic effect. These findings highlight the potential of cold compresses to provide early symptomatic relief, even though long-term benefits were not sustained.

This study has several limitations that should be considered when interpreting the results. First, the open-label design may have introduced performance or observer biases, particularly in the assessment of subjective outcomes such as pain intensity. Second, the sample size, while sufficient for detecting large effects, may have been underpowered to identify subtle differences between groups. Third, the study was conducted in a single specialized center, which may limit the generalizability of the findings to other healthcare settings with varying levels of resources or expertise.

## Conclusion

Adjunctive cold therapy showed limited efficacy, with only short-term pain reduction, while heat therapy demonstrated no significant advantage. These findings indicate that thermal therapies, though frequently used, do not provide substantial clinical benefit in mitigating envenomation-related tissue damage and inflammation. Therefore, we recommend that cold therapy be routinely used as an adjuvant therapy exclusively for pain relief in the treatment of *Bothrops atrox* envenomation, in both clinical and pre-hospital settings. Given the high prevalence of Bothrops envenomation in resource-limited regions like the Amazon, further research into evidence-based adjunctive therapies is essential. Such studies should aim to identify complementary interventions that can enhance the effectiveness of antivenom treatment, thereby improving care for patients affected by this neglected tropical disease.

## Supporting information

S1 Data Clinical and epidemiological data of patients. (XLSX)
